# Metabotropic Glutamate Receptor 5 in the Medial Prefrontal Cortex as a Molecular Determinant of Pain and Ensuing Depression

**DOI:** 10.3389/fnmol.2018.00376

**Published:** 2018-10-08

**Authors:** Geehoon Chung, Sang Jeong Kim, Sun Kwang Kim

**Affiliations:** ^1^Department of Physiology, College of Korean Medicine, Kyung Hee University, Seoul, South Korea; ^2^Department of Physiology, College of Medicine, Seoul National University, Seoul, South Korea

**Keywords:** chronic pain, depression, medial prefrontal cortex, prelimbic cortex, metabotropic glutamate receptor 5

## Abstract

Pain and depression affect one another, and this bidirectional interaction implies the existence of common or interacting neural pathways. Among the neural circuits relevant to negative affection, the medial prefrontal cortex (mPFC) is known to be involved in both pain and depression. Persistent stress from physical pain and mental distress can evoke maladaptive changes in mPFC circuits to induce depression. Conversely, the unpleasant mood condition alters mPFC circuits to distort the appraisal of aversion and make individuals vulnerable to pain. In this article, recent findings regarding mPFC in chronic pain and/or depression are reviewed, with particular focus on the metabotropic glutamate receptor 5 (mGluR5). Although the involvement of mGluR5 within the mPFC in both pain and depressive disorders has been extensively studied, there are controversies regarding changes in the activity of the mPFC during chronic pain and depression, and the functional roles of mGluR5 on altered mPFC activity. We discuss alterations in the availability of mGluR5 in the mPFC in these disorders, its role in behavioral manifestations, and its possible influence on cellular subpopulations that mediate dysfunction in the mPFC. We also propose molecular mechanisms that may cause expressional changes in mGluR5 within the mPFC circuitry.

## Introduction

Chronic pain patients often develop negative mood symptoms such as depression (Turk et al., 2010; Radat et al., 2013). Conversely, patients with depressive disorders are more susceptible to pain symptoms compared with normal individuals (Gupta et al., 2007; Wise et al., 2007; de Heer et al., 2014). The clinical manifestation of comorbid pain and depression implies that common or interacting neural circuits underlie the persistence of physical pain and negative moods. Conceptually, the long-term presence of physical pain would act as a persistent stress, and the chronic unavoidable stress would, in turn, alter the neural circuits that perceive the self-state and decide the coping strategy. Previous studies involving human subjects and animal models have revealed the critical role of the prefrontal cortex in concurrent pain and negative moods (Romero-Sandoval, 2011; Lemogne et al., 2012; Chung et al., 2017; Ong et al., 2018). The medial prefrontal cortex (mPFC) processes information about the external and internal environment to appraise the present state, predict future outcomes, and to make decisions. The stress-induced alteration of mPFC activity would change the levels of cognitive flexibility that affect the subjective perception of the self-state and following decisions impacting behavioral coping strategies.

The neural circuits within the mPFC undergo various changes during the development of chronic pain and/or depression, and these alterations play a key role in the persistence of the disorder (Lemogne et al., 2012; Marsden, 2013; Guida et al., 2015; Wang G.Q. et al., 2015; Zhang et al., 2015; Davis et al., 2016; Chung et al., 2017). The molecular alterations related to glutamatergic transmission have been of particular interest, including metabotropic glutamate receptor (mGluR)-mediated neuronal changes. Among the mGluRs, mGluR5 is one of the most studied receptor in various neurological disorders including chronic pain and mood disorders (Kolber, 2015; Pillai and Tipre, 2016; Sengmany and Gregory, 2016; Esterlis et al., 2018), given its known role in plastic changes in neural circuits (Bordi and Ugolini, 1999; Ribeiro et al., 2010). mGluR5 flexibly controls neuronal firing, and is not only responsible for physiological experience-dependent neuronal plasticity but also maladaptive changes in neural circuits which lead to neurological disorders. The activation of mGluR5 influences synaptic transmission and the intrinsic excitability of neurons. mGluR5 is densely expressed in the mPFC, and expression levels in the mPFC are altered in conditions of chronic pain or depressive disorders (Matosin et al., 2014; DeLorenzo et al., 2015; Lee K.W. et al., 2015; Chung et al., 2017; Esterlis et al., 2017).

However, there have been discrepancies in the previous reports regarding the direction of altered mPFC activity, the expressional changes of mGluR5 within the mPFC (mPFC-mGluR5), and their functional consequences. In this article, we discuss chronic pain-induced hypo- or hyper-excitability of mPFC pyramidal neurons, and their roles in pain and depression. Furthermore, we offer our perspective on the issue of contrasting reports, the specific mPFC neuronal subpopulation that may be affected by mGluR5 alteration, and possible underlying molecular mechanisms.

## Activity Changes in the mPFC in Chronic Pain States

Numerous animal model studies have found that the activity of mPFC neurons is altered in the pain state, and that the altered activity of pyramidal and/or GABAergic neurons is associated with increased pain perception. In rodents, the mPFC consists of three subregions: anterior cingulate cortex (ACC); prelimbic cortex; and infralimbic cortex. Although the role of hyperactive ACC activity on abnormal pain is well established (Rainville et al., 1997; Hsieh et al., 1999; Seminowicz et al., 2009; Koga et al., 2015; Santello and Nevian, 2015), controversies remain regarding the actions of the prelimbic and infralimbic subregions on pain perception. In this article, we primarily focus on the prelimbic subregion, as the prelimbic cortex rather than infralimbic cortex is implicated in the emotional dimension of pain (Jiang et al., 2014).

Many researchers have associated hypoactive prelimbic pyramidal neurons with increased pain perception and attenuation of pain modulatory function. Previous studies have demonstrated that pyramidal neuronal activity in the prelimbic cortex is decreased in several animal models of chronic pain, and that increasing its activity ameliorates pain. According to electrophysiological analyses, reduction of prelimbic pyramidal neuronal activity was associated with decreased intrinsic excitability of pyramidal neurons (Wang G.Q. et al., 2015; Radzicki et al., 2017), reduced excitatory (glutamatergic) inputs (Kelly et al., 2016; Cheriyan and Sheets, 2018), and increased inhibitory (GABAergic) inputs to neurons (Ji et al., 2010; Zhang et al., 2015; Kelly et al., 2016; Kiritoshi et al., 2016; Cheriyan and Sheets, 2018).

Among these mechanisms, the increased influence of GABAergic neurons on pyramidal neurons has been actively studied. The increase in inhibitory inputs to pyramidal neurons is due to the loss of available endocannabinoid in pyramidal neurons (Kiritoshi et al., 2016), and the increased activity of GABAergic neurons themselves (Zhang et al., 2015). Kiritoshi et al. (2016) found that in a model of arthritis, mGluR5-mediated production of 2-arachidonyl glycerol (2-AG) is impaired in mPFC infralimbic pyramidal neurons. Due to the lack of 2-AG, presynaptic CB1 receptor-mediated suppression of GABA release is disrupted, which leads to abnormally enhanced inhibition to postsynaptic pyramidal neuron. Considering the study of prelimbic neurons in the same model (Ji et al., 2010), this breakdown of mGluR5-endocannabinoid signaling might occur in the prelimbic pyramidal neurons as well. The increase of GABAergic neuronal activity has been reported in both of arthritic and neuropathic pain models. GABAergic neurons that inhibit prelimbic pyramidal neurons receive more excitatory signals in the chronic pain state compared with normal (Ji et al., 2010; Zhang et al., 2015). This indicates increased synaptic influences from the presynaptic glutamatergic excitatory neurons to the postsynaptic GABAergic inhibitory neurons. However, the mechanisms underlying increased synaptic transmission to GABAergic neurons remain unclear, and it has not been studied whether the intrinsic excitability of GABAergic neurons is also increased in the chronic pain state.

On the other hand, some studies have found increased mPFC neuronal activity in the pain state. These investigations showed that pharmacological deactivation of activity reduces chronic pain symptoms. In a study by Wu et al. (2016) the excitability of prelimbic layer 5 pyramidal neuron was increased in a complete Freund’s adjuvant (CFA)-induced inflammatory pain model in mice, in contrast to the reduced intrinsic excitability (with enhanced glutamatergic transmission) of the layer 2/3 pyramidal neurons in CFA model rats (Wang G.Q. et al., 2015). Matos et al. (2015) demonstrated that the intrinsic excitability of layer 2/3 pyramidal neurons located in the ACC and prelimbic cortex was enhanced in animals with neuropathic pain, due to an increase in hyperpolarization-activated, cyclic nucleotide-gated (HCN) channel activation. With the increased open probability of HCN, the manifestation of persistent firing induced by mGluR5 activation is facilitated. The administration of an HCN channel blocker decreased neuronal excitability in the slice condition, and reduced cold but not mechanical allodynia *in vivo*. Fan et al. (2018) showed that hyperresponsive prelimbic neurons are critically involved in enhanced behavioral responses to noxious stimuli after chronic pain. The previous experience of CFA-induced chronic pain enhanced prelimbic neuronal activation following formalin assaults, and inhibiting prelimbic activity reversed aggravated formalin pain. The authors suggested that increased prelimbic cortex neuronal activity might facilitate pain via increased inhibition of periaqueductal gray (PAG) neurons.

The inconsistencies indicate that the changes in synaptic transmission and intrinsic excitability are highly specific to mPFC subregion, cell type, cortical layer, the cause and the duration of the pain. In neuropathic pain models, excitability of layer 5 pyramidal neurons was reduced in the prelimbic cortex (Radzicki et al., 2017; Cheriyan and Sheets, 2018), but not infralimbic cortex (Cheriyan and Sheets, 2018). In contrast, the activation of prelimbic neurons was increased in an inflammatory pain model (Dale et al., 2018; Fan et al., 2018), with increased excitability of layer 5 pyramidal neurons (Wu et al., 2016). Interestingly, intrinsic excitability was reduced in the layer 2/3 pyramidal neurons in the same inflammatory pain model (Wang G.Q. et al., 2015). Also, it is suggestive that the changes are specific to the separate subpopulations of mPFC neurons which have connections to different brain regions (Lee M. et al., 2015; Kelly et al., 2016; Cheriyan and Sheets, 2018; Kiritoshi and Neugebauer, 2018).

Although further studies are needed to clarify apparent contradictions between a hyperactive and hypoactive mPFC in the chronic pain state, research to date has commonly observed alterations in excitatory and/or inhibitory influences on pyramidal neurons, and these disturbances are critically involved in the pain itself and the ensuing affective and cognitive disorders. Interestingly, multiple studies have described mGluR5 as a molecular mediator of altered mPFC pyramidal neuronal activity in the chronic pain state, but with inconsistent descriptions of its functional roles.

## The Effect of mPFC-mGluR5 Blockade on the Modulation of Pain and Depression

There are conflicting reports regarding the effect of mGluR5 blockade in the mPFC on pain modulation (Ji and Neugebauer, 2011; Giordano et al., 2012; Palazzo et al., 2014; David-Pereira et al., 2016; Chung et al., 2017). A previous study demonstrated that application of mGluR5 antagonist to the prelimbic and infralimbic cortex facilitated neuropathic pain induced by spared nerve injury (SNI) surgery in mice (Giordano et al., 2012). In contrast, other studies have reported that the administration of mGluR5 antagonist to the infralimbic or prelimbic cortex produced analgesic effects in an animal model of arthritis (David-Pereira et al., 2016) or spinal nerve ligation (SNL)-induced neuropathic pain (Chung et al., 2017). It is noteworthy that pain facilitation occurred when mGluR5 was blocked in the mPFC contralateral to the peripheral pain (Giordano et al., 2012), whereas pain suppression was induced when the administration was targeted to the mPFC ipsilateral to the peripheral pain (David-Pereira et al., 2016) or to the bilateral mPFC (Chung et al., 2017). Interestingly, in the SNL-induced neuropathic pain study, mGluR5 was increased in the prelimbic subregion of the mPFC ipsilateral – but not contralateral – to the peripheral nerve injury (Chung et al., 2017). The ipsilesional increase in prelimbic mGluR5 was observed in the deep layer, presumably layer 5/6, in which long-range GABAergic and transcallosal neurons are abundant (Kawaguchi and Kubota, 1997; Lee et al., 2014; Saffari et al., 2016; Anastasiades et al., 2018). This raises the unique prospect that interhemispheric and inter-layer connectivity may be involved in disease manifestation and may explain the inconsistent results between studies. The study of Kiritoshi et al. (2016) further implicates the involvement of GABAergic influences. According to the study, activation of mGluR5 in the mPFC pyramidal neuron fails to suppress presynaptic GABA release in chronic pain state due to loss of endocannabinoid signaling. Activation of mPFC-mGluR5 could induce analgesic effect only when the CB1 receptor was coactivated with a CB1 agonist treatment. Thus, the inconsistency might be stem from the differential changes in the interacting molecules which affect the modulation of synaptic transmission.

In the field of depression research, previous studies investigating non-pain depression have reported inconsistent results regarding the alteration of prefrontal mGluR5 expression. A few studies have reported lower prefrontal mGluR5 levels in patients with major depressive disorder (MDD) compared with healthy controls (Deschwanden et al., 2011; Esterlis et al., 2017), whereas other studies have reported comparable or even higher levels of prefrontal mGluR5 (DeLorenzo et al., 2015; Gray et al., 2015). This inconsistency is likely due to differences in sex, age, time of onset, and pathophysiological differences among patients (DeLorenzo et al., 2015). In fact, Gray et al. (2015) reported that female MDD patients exhibited higher levels of mGluR5 gene expression in the PFC subregion, whereas male MDD patients exhibited lower levels. In a postmortem study by Deschwanden et al. (2011) which reported lower levels of mGluR5 in the PFC of MDD patients, 80% of the subjects were male. Interestingly, a preclinical study reported that male – but not female – rats exhibited higher levels of prefrontal mGluR5 in the depressive condition induced by chronic mild prenatal stress (Wang Y. et al., 2015). Other studies have reported that male mice with mGluR5 deletion exhibit antidepressive-like behavior, suggesting that the activity of mGluR5 primarily facilitates depression (Witkin et al., 2007; Lee K.W. et al., 2015).

In contrast to such inconsistencies regarding expressional change in mPFC-mGluR5 in the depressive state, it is generally accepted that the administration of an mGluR5 antagonist exerts an anti-depressive effect (Palucha and Pilc, 2007; Pilc et al., 2008; Chaki et al., 2013; Hashimoto et al., 2013; Hughes et al., 2013; Fuxe and Borroto-Escuela, 2015; Kato et al., 2015; Lee K.W. et al., 2015; Lindemann et al., 2015; Park et al., 2015). mGluR5 antagonist treatment induces anti-depressant-like effects in animal models of stress-induced depression and chronic pain-induced depression. A recent study reported that the mGluR5 antagonist-induced anti-depressive effect is mediated via blockade of mGluR5 in GABAergic – but not glutamatergic – neurons in the mPFC (Lee K.W. et al., 2015). Lee K.W. et al. (2015) showed that mGluR5 conditional knockout in GABAergic neurons resulted in an anti-depressive effect. Conversely, mGluR5 knockout in glutamatergic neurons induced depressive-like effects. The results from these conditional knockout mice suggest that the activation of mGluR5 in the mPFC GABAergic neurons induces depression, presumably via inhibition of glutamatergic neurons. The administration of mGluR5 antagonist *in vivo* resulted in an increase in mPFC glutamatergic neuronal activity (Lee K.W. et al., 2015) and exogenous activation of mGluR5 produced GABAergic inhibition (Ji and Neugebauer, 2011), supporting the concept. Furthermore, depressive-like effects induced by glutamatergic neuronal mGluR5 knockout could be overcome by disinhibition (i.e., blocking mGluR5 in GABAergic neurons).

## The Causal Role of mPFC-mGluR5 Upregulation in Pain and Ensuing Depression

PET studies of mGluR5 in chronic pain patients have yet to be reported, and the possible differences of mPFC-mGluR5 according to sex, age, education level, and social class of pain patients are unknown. In the preclinical level, alteration of mGluR5 was investigated in the brains of male rats with neuropathic pain in a previous neuroimaging study (Chung et al., 2017). The study demonstrated that increased mGluR5 availability in the prelimbic subregion of the mPFC is responsible for amplified pain as well as depression-like behavior. The administration of mGluR5 antagonist to the prelimbic cortex of nerve-injured animals induced analgesic and antidepressant-like effects. Conversely, the naïve animals developed mechanical allodynia-like and negative mood symptoms, such as depression and anxiety, after lentiviral overexpression of mGluR5 in the bilateral prelimbic cortex. These data support a causal role for mGluR5 upregulation in the mPFC in amplified pain and negative mood symptoms.

Although the behavioral consequences of mPFC-mGluR5 upregulation have been revealed as such, the cellular roles in these circuits remain elusive. Normally, excitatory manipulation of mPFC pyramidal neuronal activity induces an analgesic effect and ameliorates pain. Administration of ionotropic glutamate receptor (iGluR) agonists to the mPFC activated the endogenous analgesic action of the PAG (Ong et al., 2018). Alternatively, mPFC pyramidal neuronal activation increased mPFC-Nucleus accumbens (NAc) activity to reduce pain perception (Lee M. et al., 2015). The action of mGluR5 activation is generally excitatory to neurons; however, there is a gap between the pain-suppressing actions of mPFC-iGluR5 activation and mPFC-mGluR5 deactivation.

One possible explanation is that increased prelimbic mGluR5 levels eventually result in a reduction in the firing of excitatory pyramidal mPFC neurons, which project to the PAG or the NAc. Previous studies have reported that the neuronal excitability of mPFC-PAG projection neurons is decreased in chronic pain states, and the increased activity of inhibitory interneurons is responsible for the reduced activity of pyramidal neurons (Zhang et al., 2015; Cheriyan and Sheets, 2018). Interestingly, glutamate stimulation elicited inhibitory inputs to mPFC-PAG projection neurons (Cheriyan and Sheets, 2018). Although the causal relationship between mGluR5 upregulation and increased inhibitory neuronal activity has not been extensively studied, the blockade of mPFC-mGluR5, or of inhibitory neuronal activity, drives mPFC circuits in the same direction, i.e., they induce the analgesic and anti-depressive effects.

**FIGURE 1:**
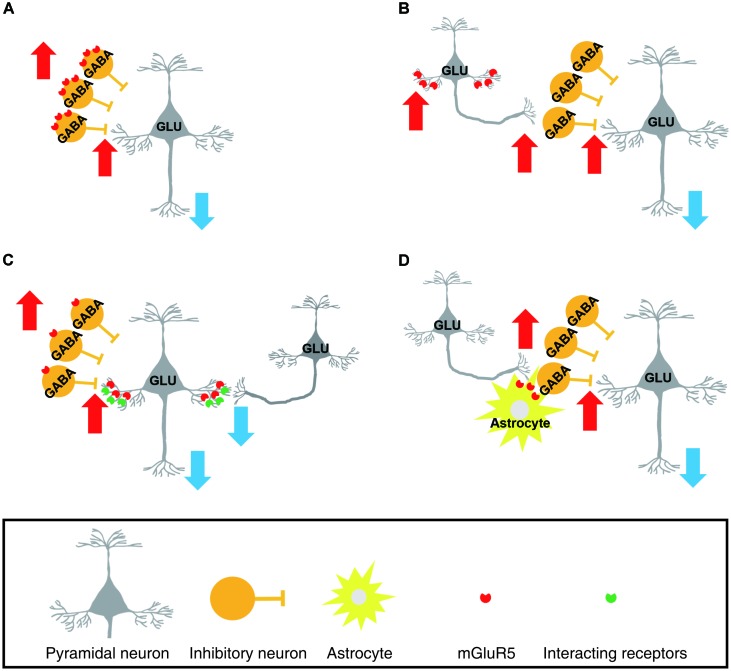
Schematic drawings representing possible cellular subsets of which metabotropic glutamate receptor 5 (mGluR5) upregulation occurs in the chronic pain state. In these scenarios, enhanced mGluR5 levels in the layer 5/6 medial prefrontal cortex (mPFC) were assumed to result in suppression of prelimbic pyramidal neurons. **(A)** mGluR5 is upregulated in the GABAergic inhibitory interneurons. As a result, GABAergic neuronal activity is increased. **(B)** mGluR5 is increased in the specific subpopulation of pyramidal neurons that preferentially excite local GABAergic neurons. **(C)** Layer 5/6 pyramidal neurons express more mGluR5. However, the increased mGluR5 suppresses, rather than facilitates, pyramidal neuronal activity. This is achieved by an enhancement of long-term depression in excitatory synapses and concurrent loss of presynaptic modulation by mGluR5. **(D)** mGluR5 upregulation occurs in astrocytes surrounding the synapses of a specific subpopulation of mPFC neurons. The increased glial mGluR5 serves to enhance excitatory influences on GABAergic neurons. (GLU, glutamatergic neuron, GABA, GABAergic neuron).

There are several conceivable hypotheses. First, mGluR5 upregulation in the chronic pain state may occur predominantly in inhibitory neurons in the mPFC (**Figure 1A**). Second, apart from this explanation, mGluR5 may be upregulated in excitatory neurons that preferentially excite inhibitory interneurons within the local circuits (**Figure 1B**). In the mPFC, the activity of local GABAergic neurons is influenced by the glutamatergic excitatory inputs they receive, and the frequency – but not the amplitude – of the excitatory transmission to the GABAergic neurons were increased in the neuropathic pain state (Zhang et al., 2015). Third, alternatively, activated mGluR5 in the pyramidal synapse may induce glutamatergic long-term depression in pyramidal neurons via interaction with other molecular signals (Otani et al., 1999; Zhong et al., 2008; Ghoshal et al., 2017; **Figure 1C**). For example, a recent study found that coactivation of mGluR5 and the M1 muscarinic acetylcholine receptor in the mPFC exerted a long-lasting decrease in excitatory transmission and concurrent enhancement of GABAergic inhibitory tone (Ghoshal et al., 2017). In this scenario, pyramidal neuronal increase in mGluR5 would not be able to modulate the presynaptic release of GABA because mGluR5 signaling in the mPFC pyramidal neurons fails to engage 2-AG mediated endocannabinoid signaling in chronic pain states (Kiritoshi et al., 2016). It has been reported that in the chronic pain state, mGluR5 activation could not increase pyramidal neuronal activity unless the inhibition from the GABAergic neuron is blocked by treatment with a GABA antagonist or activation of the CB1 receptor in GABAergic neurons (Kiritoshi et al., 2016). Fourth, mGluR5 upregulation may occur in glial cells surrounding the synapses of a specific subpopulation of mPFC neurons (**Figure 1D**). Glial mGluR5 serves to evoke complex and bi-directional effects on the modulation of neuronal activity, and upregulated mGluR5 in cortical astrocytes contributes to synaptic remodeling during the chronic neuropathic pain state (Kim et al., 2016; Ishikawa et al., 2018).

Either way, upregulated mGluR5 in the chronic pain state may be working in a direction that increases inhibitory influences on mPFC pyramidal neurons. In fact, mPFC glutamatergic neuronal activity was increased in response to the mGluR5 antagonist treatment *in vivo* (Lee K.W. et al., 2015). mPFC pyramidal neurons comprise several subtypes with different firing properties, expressing receptors, and projecting brain regions, and subserve distinct functions. Therefore, the subpopulation-specific upregulation of mGluR5 and its relevance to pain and depression should be investigated in future studies.

## Biological Processes Underlying mPFC-mGluR5 Upregulation: Possible Molecular Mechanisms

The biological processes that mediate the alteration of mPFC circuits and the cellular mechanisms underlying upregulation of mPFC-mGluR5 are largely unknown. A previous study revealed that depression is critically related to mPFC-mGluR5, and the expression level is regulated by protein p11 (Lee K.W. et al., 2015). As mentioned, the lentiviral knock-down of mGluR5 in excitatory neurons in the mPFC facilitated depressive-like behavior, whereas inhibitory neuron-specific mGluR5 knock-down exerted an anti-depressive effect. Similar results were obtained from the conditional knockout of protein p11 in excitatory or inhibitory neurons. It is worth noting that although p11 could be a strong candidate to be a mediator of mPFC-mGluR5 alteration, p11-mediated changes would predominantly occur in a specific subtype of prelimbic layer 2/3 pyramidal neurons, with decreased expression in response to chronic stress (Seo et al., 2017). Thus, the molecular mechanisms underlying upregulated mGluR5 in the prelimbic area of animals with chronic pain (Chung et al., 2017) remain vague.

A possible candidate is dopamine 2 receptor (D2R)-mediated control of mGluR5 expression. From the perspective of a reinforcement learning paradigm, dopamine signaling plays a key role in motivated approach or avoidance behavior following aversion. Because D2Rs have a strong binding affinity compared with the dopamine 1 receptor (D1R), persistent dopamine would be mainly occupied by D2R in the nervous system. When negative prediction error occurs by salient event (aversion), dopaminergic neurons would cease firing, resulting in transient dopamine depletion in the relevant brain regions (Bromberg-Martin et al., 2010; Glimcher, 2011). Theoretically, the reinforcement learning mechanisms induced by these types of negative prediction errors are to correct one’s expectancies and behaviors to avoid determinants responsible for aversion. However, some determinants, such as intractable somatic pain or psychological distress are essentially unavoidable. Long-term experiences of unavoidable, persistent distress would alter the prefrontal circuits that assess the internal state to decide coping strategies (Roy et al., 2014; Shrestha et al., 2015; Aliczki et al., 2016; Zhang et al., 2017). It is well established that chronic pain, as well as depression, leads to hypodopaminergic tone in the brain (Niikura et al., 2010; Belujon and Grace, 2017; Ong et al., 2018). Because the baseline occupancy of dopamine is higher in D2Rs, the reduction in spontaneous dopamine level predominantly affects the D2R, leading to receptor deactivation (Bromberg-Martin et al., 2010). The deactivation of D2R due to somatic pain or psychological distress has been implicated in both animal and human studies. Interestingly, D2R deactivation could increase mGluR5 availability in the striatum (Mao and Wang, 2016). According to the previous study, pharmacological deactivation of D2R resulted in an increase in mGluR5 trafficking to the synaptic membrane, which is mediated by the Src kinase family Fyn (Mao and Wang, 2016). The researchers reported that the same regulation of Fyn, which is induced by D2R deactivation, was also observed in the mPFC (Mao and Wang, 2017). Hence, it is plausible that mPFC-mGluR5 increase in chronic neuropathic pain is related to D2R deactivation, although further study is needed to elucidate the occurrence of the phenomenon in the mPFC.

## Concluding Remarks

Experiencing unavoidable stress, such as somatic pain or psychological distress, alters mPFC circuitry and distorts the perception of the self-state and ensuing decisions regarding coping strategies. We propose that mGluR5 in the mPFC may be a common mediator for both pain and depression. mGluR5 levels would regulate the gain for the assessment of internal state, guide the appraisal of the external state, and be involved in updating the expectancies of behavioral outcome in the mPFC. Increased mPFC-mGluR5 levels during the pathological state would inhibit the activity of pyramidal neurons and reduce the flexibility of the system, resulting in the loss of control of the appropriate processing of the information.

## Author Contributions

GC conceived the idea, performed the bibliographical research, wrote the draft, and revised the manuscript. SJK and SKK supervised and revised the manuscript.

## Conflict of Interest Statement

The authors declare that the research was conducted in the absence of any commercial or financial relationships that could be construed as a potential conflict of interest.
